# Whither Magnetic Hyperthermia? A Tentative Roadmap

**DOI:** 10.3390/ma14040706

**Published:** 2021-02-03

**Authors:** Irene Rubia-Rodríguez, Antonio Santana-Otero, Simo Spassov, Etelka Tombácz, Christer Johansson, Patricia De La Presa, Francisco J. Teran, María del Puerto Morales, Sabino Veintemillas-Verdaguer, Nguyen T. K. Thanh, Maximilian O. Besenhard, Claire Wilhelm, Florence Gazeau, Quentin Harmer, Eric Mayes, Bella B. Manshian, Stefaan J. Soenen, Yuanyu Gu, Ángel Millán, Eleni K. Efthimiadou, Jeff Gaudet, Patrick Goodwill, James Mansfield, Uwe Steinhoff, James Wells, Frank Wiekhorst, Daniel Ortega

**Affiliations:** 1 IMDEA Nanoscience, Faraday 9, 28049 Madrid, Spain; irene.rubia@imdea.org (I.R.-R.); antonio.santana@imdea.org (A.S.-O.); francisco.teran@imdea.org (F.J.T.); 2 Geophysical Centre of the Royal Meteorological Institute, 1 rue du Centre Physique, 5670 Dourbes, Belgium; simo@meteo.be; 3 Soós Water Technology Research and Development Center, University of Pannonia, 8200 Nagykanizsa, Hungary; e.tombacz@chem.u-szeged.hu; 4 RISE Research Institutes of Sweden, Sensors and Materials, Arvid Hedvalls Backe 4, 411 33 Göteborg, Sweden; christer.johansson@ri.se; 5 Instituto de Magnetismo Aplicado UCM-ADIF-CSIC, A6 22,500 km, 29260 Las Rozas, Spain; pmpresa@ucm.es; 6 Departamento de Física de Materiales, Universidad Complutense de Madrid, Avda. Complutense s/n, 28048 Madrid, Spain; 7 Nanotech Solutions, Ctra Madrid, 23, 40150 Villacastín, Spain; 8 Department of Energy, Environment and Health, Instituto de Ciencia de Materiales de Madrid (ICMM/CSIC), Sor Juana Inés de la Cruz 3, 28049 Madrid, Spain; puerto@icmm.csic.es (M.P.M.); sabino@icmm.csic.es (S.V.-V.); 9 UCL Healthcare Biomagnetics and Nanomaterials Laboratories, 21 Albemarle Street, London W1S 4BS, UK; ntk.thanh@ucl.ac.uk; 10 Biophysics Group, Department of Physics and Astronomy, Gower Street, London WC1E 6BT, UK; 11 Department of Chemical Engineering, University College London, Torrington Place, London WC1E 7JE, UK; m.besenhard@ucl.ac.uk; 12 Laboratoire Matière et Systèmes Complexes MSC, Université de Paris/CNRS, 75013 Paris, France; claire.wilhelm@univ-paris-diderot.fr (C.W.); florence.gazeau@univ-paris-diderot.fr (F.G.); 13 Endomag, The Jeffreys Building, St John’s Innovation Park, Cowley Road, Cambridge CB4 0WS, UK; qharmer@endomag.com (Q.H.); emayes@endomag.com (E.M.); 14 Biomedical Sciences Group, Translational Cell and Tissue Research Unit, Department of Imaging and Pathology, 3000 Leuven, Belgium; bella.manshian@kuleuven.be (B.B.M.); s.soenen@kuleuven.be (S.J.S.); 15 INMA Instituto de Nanociencia de Materiales de Aragón, Pedro Cerbuna 12, 50009 Zaragoza, Spain; yuanyugu@hotmail.com (Y.G.); amillan@unizar.es (Á.M.); 16 Chemistry Department, Inorganic Chemistry Laboratory, National and Kapodistrian University of Athens, Panepistimiopolis Zografou, 15771 Athens, Greece; e.efthimiadou@inn.demokritos.gr; 17 Magnetic Insight, Alameda, CA 94501, USA; gaudet@magneticinsight.com (J.G.); goodwill@magneticinsight.com (P.G.); jmansfield@magneticinsight.com (J.M.); 18 Physikalisch-Technische Bundesanstalt, Abbestraße 2-12, 10587 Berlin, Germany; uwe.steinhoff@ptb.de (U.S.); james.wells@ptb.de (J.W.); frank.wiekhorst@ptb.de (F.W.); 19 Institute of Research and Innovation in Biomedical Sciences of the Province of Cádiz (INiBICA), 11002 Cádiz, Spain; 20 Condensed Matter Physics Department, Faculty of Sciences, Campus Universitario de Puerto Real s/n, 11510 Puerto Real, Spain

**Keywords:** magnetic hyperthermia, magnetic nanoparticles, hysteresis losses, cancer, magnetic particle imaging, theranostics, nanoparticles synthesis, thermometry, standardization, nanotoxicity

## Abstract

The scientific community has made great efforts in advancing magnetic hyperthermia for the last two decades after going through a sizeable research lapse from its establishment. All the progress made in various topics ranging from nanoparticle synthesis to biocompatibilization and in vivo testing have been seeking to push the forefront towards some new clinical trials. As many, they did not go at the expected pace. Today, fruitful international cooperation and the wisdom gain after a careful analysis of the lessons learned from seminal clinical trials allow us to have a future with better guarantees for a more definitive takeoff of this genuine nanotherapy against cancer. Deliberately giving prominence to a number of critical aspects, this opinion review offers a blend of state-of-the-art hints and glimpses into the future of the therapy, considering the expected evolution of science and technology behind magnetic hyperthermia.

## 1. Introduction

The scientific community involved with magnetic hyperthermia may be on the verge of another turning point after some years without relevant news on the outcomes of clinical research: new clinical studies on different indications are currently taking place. For example, MagForce AG recently announced that its American subsidiary MagForce USA, Inc. obtained approval from the U. S. Food and Drug Administration (FDA) for a pivotal single-arm study for the focal ablation of intermediate-risk prostate cancer with their NanoTherm^®^ therapy system [1]. In Europe, both the Vall d’Hebron University Hospital and the Fuenlabrada University Hospital are home to a new feasibility study on treating locally advanced pancreatic ductal adenocarcinoma (PDAC) within the remit of the NoCanTher project [2].

Without any doubt, behind the progress so far on the clinical translation of magnetic hyperthermia, there is an ever more intertwined scientific network worldwide that is keeping a constant influx of basic research, consolidating the developments under the light of consensual new procedures, and expanding links with key actors in the translational and clinical arena. International networking initiatives, such as the “RADIOMAG” COST action [3], have helped in fighting against the geographical dispersion of scientific and human resources related to magnetic hyperthermia, as well as eliminating duplication of research lines and contributing to the harmonization of key concepts and procedures. In any case, the cooperation between clinical and non-clinical worlds has become much more fluid, as it should be to achieve a sustainable improvement in the coming decades [4]. The existence of unique infrastructures for reliable, dedicated and widespread characterization techniques for nanomedicines is paving the way for a faster translation of promising nanoproducts. A supranational example is the European Nanomedicine Characterisation Laboratory [5], created back in 2015 under the auspices of the H2020 framework program, and a more established national example is the Nanotechnology Characterization Laboratory in the USA, founded by the National Cancer Institute (NCI) in collaboration with the FDA and the National Institute of Standards and Technology (NIST) [6]. However, as it could not be otherwise, there are some important issues standing in the way of wider clinical adoption of magnetic hyperthermia, some of which are common to many other nanomedicines [7]. The economic burden of taking the leap from basic nanomedicine research to translation [8] appears to be insurmountable in the opinion of the scientific community, above all with the current funding schemes, which despite being regarded as insufficient and poorly coordinated, are also beginning to suffer significant cuts. This is exemplified by the recent turmoil around the decision of the United States National Cancer Institute (NCI) in halting funding for the Centers of Cancer Nanotechnology Excellence (CCNEs) [9], the commentary published by Kinam Park—the former Editor-in-Chief of the Journal of Controlled Release—in favor of the controversial decision [10], and the counterreaction that followed from the board of the Nanomedicine and Nanoscale Delivery Focus Group of the Controlled Release Society [11] and the former president of the European Research Council [12].

Magnetic hyperthermia therapy mainly comprises two key elements: injectable magnetic nanoparticles (MNPs) and a magnetic field applicator, both of which were approved in most cases as medical devices. At least in Europe, there is still a debate around whether a more specific regulatory framework—beyond the more recent regulation (EU) 2017/745 on medical devices repealing the 93/42/EEC and 90/385/EEC directives—is needed for nanomedical devices. The matter only worsens when considering the process in different pharmaceutical jurisdictions [13]. This uncertainty around well-defined pre-normative and regulatory frameworks is discouraging private investors and pharmaceutical companies from taking the initiative in leading new industrial projects or sponsoring the most promising current developments. Added to this is the reluctance to use MNPs in humans after several cases of withdrawals of nanoproducts both from the market and from the regulatory process, in addition to the abandonment of the production of other formulations based on MNPs (see Section 5).

All these aspects, along with many others shaping the present and the future of magnetic hyperthermia, are commented on here by international experts, taking the current state-of-the-art as a starting point.

## 2. Establishing Standard Operational Procedures for Structural and Magnetic Characterization of Magnetic Nanoparticles

Nowadays, nanomaterials manufacturers face a constant increase of requirements regarding speed process and product quality control that need real-time characterization techniques adapted to nanoscale metrology and standardized operational procedures (SOP). Indeed, SOP and automated instrumentation for characterizing magnetic nanomaterials will definitively benefit both the industrial demands in quality control and also basic research. Recent initiatives, as the “RADIOMAG” COST action [3], showed significant variability of results when comparing physical parameters, such as the specific absorption rate (SAR) or specific loss power (SLP), obtained in magnetic colloids by different research labs [14]. Moreover, many physical parameters of MNPs can be determined by distinct characterization techniques (see Table 1), increasing the variability of the results. Hence, there is a need for standardizing methodologies for characterizing extremely relevant parameters such as magnetic losses of MNPs.

Since this section focuses only on essential characterization techniques for magnetic hyperthermia (MH) applications, it is worth noting that magnetic losses are strongly influenced by MNPs parameters such as size [17] and shape [18,19], aggregation degree [20,21] magnetic anisotropy [22], magnetic dipolar interactions [23,24], functionalization [25,26], viscosity of the dispersion medium [27,28], and alternating magnetic field conditions (field frequency and amplitude) [29,30,31]. Several EU projects focused on standardization and harmonization of analysis methods for MNPs have been/are being carried out, e.g., NanoMag, MagNaStand and RADIOMAG, as well as approved ISO standards (ISO/TS 19807-1:2019) [32,33]. These achievements benefit the preparation of SOPs for characterizing relevant parameters such as magnetic losses of MNPs or the design of standard reference nanomaterials to harmonize the comparison of results obtained by distinct research groups. Hence, SOPs aim to homogenize procedures for characterizing physicochemical parameters of magnetic suspensions as the first step towards International Standards. So far, efforts with good results have been done to characterize and harmonize analysis methods for both suspended and immobilized MNPs [15,16,33,34]. Here we spotlight selected essential methods for MH application. A general description of analysis methods for magnetic nanoparticle systems can be found in ref. [16].

### 2.1. Structural Characterization

Today, nanoscience cannot exist without near-field and electron microscopy techniques such as TEM, HRTEM, SEM, EDX, AFM, etc. Within the latter, TEM is the most widely used for the structural characterization of nanoparticles, which mainly comprises MNP core size, core size distribution, shape, aggregation, etc. However, due to the inherent sample preparation techniques, it is often difficult to preserve the original colloidal state. In this sense, the use of cryo-TEM is encouraged to better capture the spatial arrangement of MNPs, thus providing more accurate information about their aggregation state.

### 2.2. Colloidal Properties

These are generally characterized under random conditions, namely pure water, buffers (often phosphate solutions), etc. A priori well-qualified samples, however, often end up failing in vivo due to a significant loss of efficacy and/or the onset of toxicity [35]. The reasons behind this observation can be diverse: sample contamination, MNPs aggregation, and interfacial interactions with cell membranes or blood components, among others [36]. MNPs qualification must be performed under conditions that mimic the in vivo environment, mainly pH and salinity, but also including proteins, carbohydrates and lipids. In general, MNPs’ interactions at bio-nano interfaces are mainly determined by size, charge and hydrophilicity/hydrophobicity [37]. In fact, these properties are closely related to the parent colloid stability—via electric, steric and electrosteric stabilization—and particle aggregation in poorly stabilized magnetic fluids. Dynamic light scattering (DLS) is one of the most employed methods to measure hydrodynamic sizes and size distributions in dilute colloids by analyzing the intensity fluctuation of scattered light caused by the Brownian motion of the constituent nanoparticles. The main source of uncertainty here is polydispersity, but in the literature, the “DLS size” is often provided without reporting some relevant measurement conditions like pH or ionic strength, making it difficult to establish the source of polydispersity. The latter could reside in the primary particles and their aggregation due to weak colloidal stability [35], and it has a major impact both on the sample’s shelf life and its subsequent use. For example, appropriate and inappropriate MNP manufacturing has been illustrated in the literature by human blood smear tests [36].

MNP charge can be characterized via zeta potential (ζ) measurements, which is not characteristic of surface charge as found in the literature [15]. It highly depends on the pH and ionic strength of the medium and the quality and quantity of specific ions (phosphates in buffers, carboxylates, surfactant ions, etc.). If MNPs are stabilized only electrostatically, ζ values higher than |25–30| mV, measured at low ionic strength, indicate good colloidal stability. At high salt concentration, ζ becomes zero. A null value also occurs both at a pH coinciding with the isoelectric point and in the presence of specific ions, causing ζ reversal. Consequently, reporting ζ values without providing information on pH, ionic strength, specific ions, etc., of the dispersion solutions is meaningless. In the case of concentrated magnetic suspensions and gels, DLS cannot be used; in these cases, more powerful scattering methods such as SAXS and SANS are needed [38]. The core-shell structure and the probability of aggregation in samples can be measured in pristine samples as used in bio-relevant media, even highly concentrated or embedded in a gel. The third relevant colloidal parameter is the hydrophilicity/hydrophobicity of the MNP coating. Of particular note in the case of MNPs intended for biological media is the protein adsorption, leading to the so-called “protein corona” around nanoparticles since it masks the original character of the MNP surface [35].

### 2.3. AC Susceptometry

In AC susceptibility (ACS) vs. frequency measurements, a sinusoidal magnetic field of constant amplitude is applied over the sample, and the excitation frequency is swept at a constant temperature [39,40,41,42]. A superimposed DC magnetic field can also be applied. The AC field intensity is generally sufficiently small, fulfilling the low-field limit where the magnetization is linear to the field. The in-phase component (real part) and out-of-phase component (imaginary part) of the ACS are measured versus excitation frequency. In order to calibrate the signal amplitude and phase, the system should be calibrated, e.g., with a sample with a known dynamic magnetic frequency response, for instance, the paramagnetic material Dy_2_O_3_ in powder form [43]. This also allows to compensate for any amplitude and phase errors and also to convert the measured ACS into a calibrated volume, molar or mass susceptibility. ACS vs. frequency measurements have been routinely used by numerous groups to characterize MNPs [43,44,45,46]. From the ACS response, it is possible to estimate the SLP value by studying the magnetic losses obtained from the ACS out-of-phase component [41,47].

In AC susceptibility vs. temperature measurements at constant excitation frequency, a small amplitude sinusoidal magnetic field is also used, and its frequency can be varied up to about 10 kHz [48]. In a recent paper, an induction-based ACS system that can be used at lower temperatures was designed for frequencies up to the MHz range [49]. Calibration is done in almost the same way as the ACS vs. frequency method using a sample with known dynamic magnetic properties. The in-phase and out-of-phase components of the ACS are measured versus the temperature of the sample. In addition, in this case, a superimposed DC magnetic field can be applied. In a specific temperature range, the response becomes frequency-dependent, and the ACS results provide information about the magnetic relaxation properties of the MNP ensemble [49,50,51,52,53,54,55]. Thus, measuring the dynamic magnetic properties gives information on the magnetization dynamics in the sample by varying the AC drive frequency (different time scales). Temperature-dependent ACS is a standard technique for characterization of MNPs, for instance, to determine blocking temperatures, magnetic relaxation properties or magnetic interactions; indeed, it is important to quantify magnetic interactions as they will affect the energy absorption and, therefore, the hyperthermia heating properties [17,56,57,58].

### 2.4. DC Magnetization

In DC magnetometry (DCM), the magnetic moment of a sample is measured as a function of both applied magnetic field and temperature. DCM measurements are typically performed in commercially available magnetometers, based on SQUID techniques, vibrating sample magnetometers (VSM) or alternating gradient magnetometers (AGM) [59]. The maximum magnetic fields in the DCM method should be large enough to saturate the sample magnetization in order to determine the intrinsic saturation magnetization. DCM magnetometers are calibrated against a magnetic sample with known saturation magnetization or susceptibility. The basic parameters from a magnetization versus field are intrinsic saturation magnetization, where the measured magnetic moment is normalized to the mass or volume of the magnetic material under investigation. In addition, the remanence and coercivity from the hysteresis loop can be determined. Likewise, the absorbed energy by the MNP system at equilibrium can be obtained by calculating the area enclosed under the hysteresis loop [60]. DC magnetization measurements constitute a basic magnetic characterization technique that has been routinely used by numerous groups to characterize MNPs systems [61,62,63,64].

### 2.5. AC Calorimetry

Calorimetry is the most employed technique to quantify magnetic losses in MNP suspensions subjected to an AC field. The procedure is based on measuring the initial temperature increase rate immediately after applying the AC field. This experimental method has been widely employed and has contributed to understand the influence of intrinsic—structural, colloidal, magnetic—or extrinsic parameters—AC field—on SLP and ILP values [20,23,24,26,30,31,36,47,56,57,58,63,65,66,67,68]. Calorimetric measurements are usually performed under non-adiabatic conditions since adiabatic ones are rarely attained [69,70]. Such non-adiabatic systems require particular data analysis to remove artifacts from different error sources [71,72].

### 2.6. AC Magnetometry

AC magnetometry quantifies the enclosed area of AC magnetic hysteresis loops to determine SLP values (≈area under loops × field frequency). The application of this technique to measure magnetic colloids is recent, and most of the obtained results have been performed using home-made equipment [73,74,75,76] since commercial equivalents are very scarce. AC magnetometry has the advantage that the calculation of SLP values is not influenced by thermal parameters or conditions, allowing to quantify of magnetic losses when MNPs are inside biological matrices, like cells or tissues [28]. The analysis of hysteresis loops under AC fields can shed light on the effect of particle size, shape, aggregation, anisotropy, viscosity and field amplitude and frequency on the magnetic losses [27,28,77,78,79]. However, dedicated SOP are also needed for this technique.

In summary, the existence of SOP and automated instrumentation to quantify relevant physicochemical parameters to magnetic hyperthermia will warrant the reliability and reproducibility of the obtained values, which is mandatory to ensure a reliable translation of MH to clinics.

## 3. Scalable Synthesis Protocols

### 3.1. General Challenges

Today’s literature provides a variety of protocols to synthesize uniform ferrite MNPs with different sizes and shapes, suitable for magnetic hyperthermia [80]. In many cases, reported nanoparticle properties are superior to those of currently approved products and, therefore, have the potential to increase the efficiency of hyperthermia treatments by reaching higher temperatures at lower nanoparticle concentrations under milder magnetic field conditions. However, large-scale production of these MNPs with improved or optimal properties is associated with obstacles such as low yield and, most importantly, limited reproducibility due to poor control and documentation of synthesis conditions. These challenges need to be addressed for a synthetic product to reach market maturity.

On the other hand, a current research challenge is understanding nanoparticle formation mechanisms and kinetics that are essential to guide the development of syntheses that are reproducible in a systematic, controllable and scalable manner. Since continuous processes can provide advantages over batch processes for reproducible and scalable synthesis protocols, they have recently gained increased interest.

The final step related to nanoparticle functionalization is still a difficult task that needs special attention to achieve scalable production. The coating and the number of active surface sites are crucial for nanoparticle dispersion/stability, and therefore particle–particle magnetic interactions and the particle heating properties independently on the media viscosity and the concentration, i.e., in vitro and in vivo conditions. It is important to establish a reproducible yield of the coating step and purification of any byproduct.

### 3.2. Preparation of MNPs and Functionalization

We have identified some major challenges in the preparation of uniform MNPs with different synthetic methods, classified by the media where nucleation and growth take place, i.e., aqueous and polar or nonpolar organic solvents. We focus on magnetic iron oxide nanoparticles (IONPs) as they have already been approved for humans [81] and are therefore the most promising heating agent candidates.

The main challenge of synthesizing IONPs for hyperthermia in aqueous media is the production of large (~20 nm) particles with good control of size and shape distribution. Despite progress and broad utilization of the water-based co-precipitation method due to the high yield, there are several drawbacks, such as suboptimal size (<15 nm, with exceptions, e.g., for methods with a slow pH increase [82]), high polydispersity, poor crystallinity control, and consequently poor saturation magnetization. Regarding larger particles for hyperthermia application with saturation magnetization values near the bulk, they can be obtained by oxidative precipitation of Fe(II) salts in aqueous media (>15 nm). Recently, oxidative precipitation has been scaled up to 20 g per batch [83] and was made a continuous process [84,85]. The combination of low-cost reagents such as FeSO_4_, NaOH, NaNO_3_ and ethanol/water mixtures was shown to yield uniform nanoparticles.

The main challenge for thermal decomposition synthesis in high boiling point organic media (commonly nonpolar solvents such as 1-octdecene or polar polyol solvents) is the standardization of experimental procedures and control of synthetic conditions. This is due to the complexity of the formation process of magnetite and the consequence of a set of thermally activated chemical reactions [83]. Even subtle changes of chemical aspects such as precursor/reagent concentration can provoke considerable modifications of the decomposition and or nucleation temperature with dramatic consequences on the final nanomaterial product.

One parameter deserving special attention is the heating rate, as it is known to affect (or used to control) nanoparticle sizes [86]. Since the heating rate can vary with the experimental procedure, for example, the size and geometry of the reaction vessel (with limits for larger volumes in terms of heat input), reproducing heating profiles from small to especially large-scales is not trivial. Temperature profiles of the reaction media should therefore be documented carefully; reporting heater settings is not enough. Considering scalable production, efficient heating sources are required to facilitate homogeneous temperature profiles (here, the mixing system plays an important role) and sufficient heating rates. Heating via microwave radiation is a promising alternative to classical heaters and was shown to enable scale up the production up to 1 kg [87]. Microwave heating was also used to synthesis of flower-like multi-core IONPs with good control over core sizes and fusion between them depending on the reagents, temperature and heating time. Other heating systems such as hydrothermal using autoclaves presents the drawback of needing a special pilot plant to scale up safely the lab-scale procedure.

In addition to heating rates, also reagent storage, purity and supplier, stirring conditions (stirring speed, type and dimension of propeller/stirrer used), inert/non-inert gas conditions during synthesis [88] and washing/centrifugation protocols need to be documented and/or recorded accurately during synthesis to obtain good reproducibility. Only accurate documentation, and reporting, allows researchers in other laboratories to run a thermal decomposition synthesis “in the same way”, i.e., at best possible degree of similarity.

Although (classical) thermal decomposition syntheses in nonpolar solvents can yield magnetic particles with excellent size and shape uniformity, as well as crystallinity and magnetization, they are very high in price, especially due to expensive organic precursors, and generate many byproducts. Polyol methods are usually cheaper, but scalable protocols to synthesize particles for magnetic hyperthermia are still at the research stage.

Functionalization is another significant aspect, which draws interest when aiming to use nanomaterials for magnetic hyperthermia. Much work has been done in this aspect, and the protocols for coating the nanoparticles with layers of organic or inorganic agents and their functionalization depending on the application are generally well-established [89]. Their limitations are well-known and often related to the large polydispersity that is generated by the coating of aggregates, the limited colloidal stability and its degradation when stored for a long time. Most protocols use highly diluted suspensions and are difficult to scale up. A special problem that needs more attention is related to the overall influence of the coatings on the hyperthermia performance in vitro and in vivo.

### 3.3. Improving Reproducibility

In order to improve the reproducibility of all MNP synthesis, we need to develop detailed reports of methods and procedures, including used chemical reagents (e.g., purity, supplier, and product code), as well as SOPs that should be published as supplementary info with the paper to be able to become bench-mark syntheses for the community. In the same way, accurate reporting of characterization protocols is essential. The use of ISO standards that are internationally agreed by experts and guaranteed quality requirements (e.g., analysis of TEM images, usage of Scherrer equation, DLS for size, ζ-potential, VSM-SQUID for magnetic properties, TGA for the determination of the amount of organic material at the particles surface, ICP-OES elemental analysis for pure metal content quantification) should be used [15]. A good practice is the publication of raw data (especially TEM images, where an image is not presentative due to a limited number of particles that can be observed). In fact, in many cases, there is a need to use a combination of different characterization techniques in order to get a full detailed and reliable picture of a given sample. Already published ISO standards can help in the comparison of the results and the samples.

In the same sense, the development of broadly accepted protocols for the magnetic characterization of hyperthermia-targeted nanoparticles will help to provide a higher degree of reliability of hyperthermia measurements at an internationally accepted level. In this way, the measurement of both SLP and ILP (to compare measurements performed at different frequencies and field strengths or using different instruments) will be more easily comparable among all academic and non-academic partners. These characterizations will concern all possible forms, such as colloidal dispersions/ensembles and powder samples. Detailed characterization of the nanoparticles is of outstanding importance for establishing a reproducible synthesis.

### 3.4. Scalability Possibilities

Production at larger scales can easily be achieved due to repetitive and parallel production, but syntheses using lab-scale reactors (<100 mL) will hardly be cost-effective. Only in few cases, the solubility of reagents allows the large production by increasing the reagent concentration while keeping the same solvent volume [83]. In most cases, keeping the precursors/surfactants/solvents ratio while scaling-up a given reaction is not enough to assure its reproducibility in those larger scales unless demonstrated. It should be taken into account that the gap between the ordinary laboratory scale (100 mL) and the industrial pilot plant scale (100 L) is three orders of magnitude. Experiments of scaling up to 10 L that still could be done in the lab are needed before going to the industrial scale.

The key for a successful scale-up is to understand the synthesis’ critical process parameters, i.e., parameters with a high impact on the nanoparticles’ critical quality attributes (for example, in the case of hyperthermia, particle size distribution, surface functionalization, and most importantly, the SLP). Hence, understanding nanoparticle formation mechanisms and kinetics are important as it shows, for example, if mixing times, heating rates, or reagent addition time scales (or a combination) is crucial, i.e., it needs to be maintained at larger scales.

**Figure 1:**
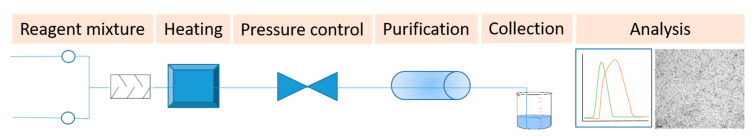
Schematic representation of a continuous flow setup for large-scale production of magnetic nanoparticles.

An interesting option to produce MNPs at larger scales is the continuous syntheses at low [85,90,91,92] and high temperatures [93,94,95,96] (Figure 1). Both processes can be automatized at a laboratory scale, but up to now, none of them have been passed to an industrial pilot plant scale.

Changes in the manufacturing process can significantly change particle properties such as size, shape, and purity. Therefore, robust nanoparticle production routes need to be chosen. It is important to select those solvents of higher chemical stability to avoid their degradation. Of utmost importance for scalable processes (and certainly when translating batch processes to flow) is the required reaction time. For practical reasons, highly exothermic reactions are undesirable, although flow reactors provide a safer alternative as they facilitate rapid heat exchange.

Regarding the scale-up of the functionalization step, the common need for stirring may complicate the attempts to scale up such experiments when applying a continuous flow process instead of “batch” ones. It needs further studies to translate the synthesis and functionalization of MNPs in continuous or segmented flow reactors for production at industrial scales.

Besides the requirement of having a robust and reproducible process, what to consider as large-scale production should be put into perspective of demand and value of the product. Assuming that a conservative estimate 0.05% of the world’s population (8 billion) would suffer from cancer that can be treated via magnetic hyperthermia, and assuming further, that each patient will need six cycles of treatment a year requiring each time 0.3 g of MNPs [97], the global demand would be 144 tons a year. We need to improve the heating efficiency of IONPs, let us say three times more, to reduce the amount of MNPs needed for each time to 0.1 g. Although this production rate appears challenging, it must be considered together with the production costs. Assuming that costs of $100 per treatment with the nanoparticles are acceptable, a reasonable cap for production costs might be $50/g (5% of treatment costs). Although this is an extremely oversimplified estimation, it indicates what can be considered as an economic large-scale synthesis of MNPs for hyperthermia, i.e., a process capable of reproducibly producing the desired nanomaterial at costs < $50/g. In biomedical applications, when a small amount of nanoparticles is needed, an alternative to scaling the production of MNPs could be the translation of the synthetic process towards a dose-on-demand synthesis in the clinic [98,99].

## 4. Long-Term Stability and Biodistribution of Nano-Heaters in Humans

One key issue in the use of magnetic nanoparticles for magnetic hyperthermia therapy is their biodistribution, biotransformation and long-term fate in the body.

A first concern is to define the time window of magnetic hyperthermia efficacy, i.e., how long the particles will keep their magnetic properties and will be able to heat. The subsequent clinical challenge is to define the number and the time of magnetic field applications that will be useful to affect the tumor.

It has been shown that intracellular confinement of magnetic nanoparticles in lysosomes has a dramatic impact on their dynamical magnetic properties and SLP, mostly due to dipolar interparticle interactions and loss of rotational mobility [100,101], even if the particles keep their crystalline integrity. Another critical aspect is that the intracellular magnetic particles are exposed to the harsh environment of lysosomes that combine acidic pH (about 4.5), enzymes that regulate protein degradation and redox regulators. Since the function of lysosomes is to degrade undesirable proteins and xenobiotics, it is important to determine to what extend lysosomes may be able to degrade magnetic particles and make them lose their structural integrity and magnetic properties?

**Figure 2:**
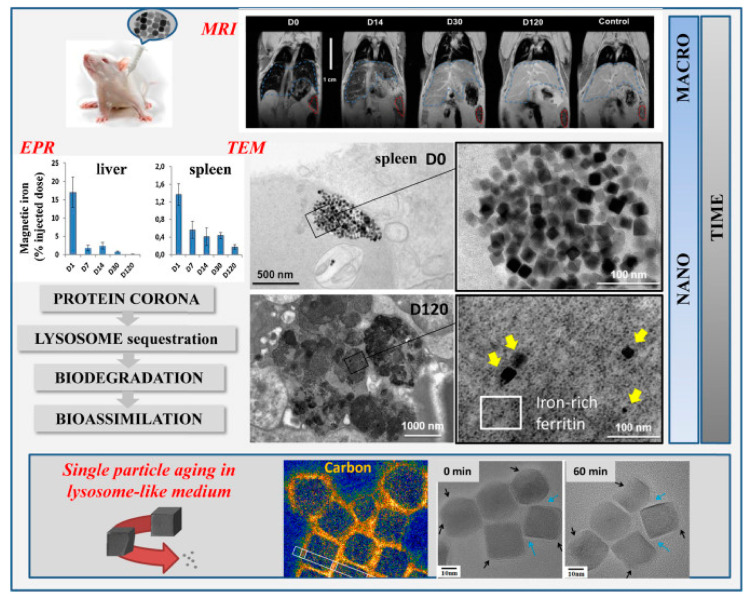
Multiscale follow-up of iron oxide nanocubes over time using in vivo magnetic resonance imaging (MRI) in mice, ex vivo electron paramagnetic resonance (EPR) quantification in organs, TEM observations of intracellular distribution and morphological biotransformations (reproduced with permission from [104].

In vivo studies in mice revealed that intravenously injected IONPs lose their magnetic properties once accumulated in the liver and spleen over months after injection [102,103]. In this regard, it was critical to develop nanometrology methods to quantify the magnetic properties of nanoparticles in organs over time after their administration. Elemental analyses such inductively coupled plasma mass spectrometry(ICP-MS) or inductively coupled plasma atomic emission spectroscopy (ICP-AES) as ICP-MS or ICP-AES measure the total iron concentration in a sample but cannot distinguish between endogenous iron—naturally present in the body—and exogenous iron coming from injected nanoparticles and between magnetic and non-magnetic iron. In contrast, magnetization measurements or ferromagnetic resonance (FMR) (also called electron paramagnetic resonance (EPR)) can be adapted to tissue samples to specifically quantify the magnetic properties in organs or cells and identify the evolution of the magnetic behavior of nanoparticles in different tissues [102] (Figure 2). Although these metrology methods provide quantitative information on the global magnetic properties of a tissue sample (loss of magnetization or modification of magnetic dynamics can be due both to degradation of nanoparticles and/or leak/transfer to other tissues), direct observations at the nanoscale are also required to assess in situ the structural biotransformation of nanoparticles in their microenvironment. Remarkably, the first non-ambiguous proof that IONPs are locally disintegrated within lysosomes in vivo was provided using iron-oxide-coated-gold nanoparticles [103]: iron oxide crystalline shell was shown to disappear around the gold core within lysosomes of spleen and liver cells. Interestingly the degradation kinetics depend on the initial coating of nanoparticles, shape, size and dose injected [103,104,105]. Iron oxide nanocubes, for example, are first eroded starting from the corners of the cube, where the polymer layer is the less dense [106] (Figure 2). Eroded nanoparticles preserve their crystalline structure. In vivo experiments also shed light on the recycling process of iron originating from the degradation of nanoparticles. This answers the second concern of nanoparticle administration in the body: what is the long-term fate of the magnetic particles? What about the product of degradation? TEM observations from in vivo tests with mice revealed the increased presence of iron-filled ferritin protein inside and outside the lysosomes, close to intact or degraded nanoparticles [102,107]. This raised the hypothesis that ferritin, the protein in charge of iron storage and regulation, could accumulate iron coming from the degradation of nanoparticles, which is consistent with the overexpression of ferritin-related genes a few months after nanoparticle injection. In vitro studies have indeed demonstrated that apoferritin has not only the ability to accumulate iron coming from the degradation of nanoparticles in an acidic lysosomal like environment but that it can also regulate the nanoparticle degradation and iron recycling, which is consistent with the role if redox regulator of ferritin inside the cells [108]. Moreover, cobalt was also detected in ferritin proteins localized in lysosomes of liver tissue in mice injected with cobalt ferrite nanoparticles [109]. This highlights that endogenous ferritin proteins can locally accumulate metal ions originating from the lysosomal degradation of nanoparticles, including at least iron and cobalt. Ferritin recycling and regulation of nanoparticle degradation in lysosomes can be a strategy to limit the toxicity of free iron ions mediated by the Fenton reaction. Hence, the finely regulated iron metabolism in the organism is a crucial asset for the clinical use of IONPs, which benefit from physiological iron ferritin storage to avoid toxicity at least at low doses.

**Figure 3:**
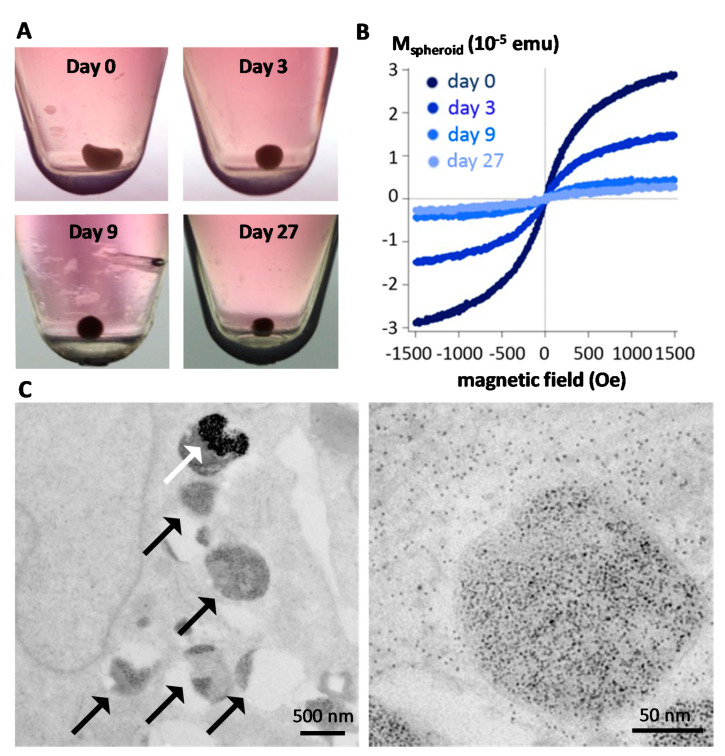
Intracellular biodegradation of MNPs monitored within cell spheroids as tissue model. (**A**) The spheroids are formed from 200,000 stem cells, which spontaneously regroup into a cohesive spherical aggregate that can be kept in culture over a month without experiencing any cell mortality or tissue necrosis. (**B**) Magnetometry can be performed at the single spheroid level, evidencing a massive degradation of the nanoparticles in a few days after internalization. (**C**) TEM images one month after nanoparticle internalization, demonstrating that only a few intact nanoparticles remain within the endosomes (white arrow) while both endosomes and cytoplasm are filled with the ferritin protein (black arrows) containing the iron released from degradation, with a diameter 5–7 nm as seen in the close-up image on the right. Reproduced with permission from [111].

Although in vivo studies have been instructive to reveal the long term fate of magnetic particles, methods to monitor quantitatively their structural integrity remained challenging to implement in situ, within the cells, and in the long-term [110]. One emerging approach is to use cell spheroids [111,112], which stands as a closed system allowing them to be kept in culture for long (up to a few months for stem cells-based spheroids, Figure 3A), and harboring nanoparticles within each of the component cells, internalized right before spheroid formation. Multiscale physical measurements (magnetism, calorimetry, optics) can be performed quantitatively at the single spheroid level and qualitatively by imaging at the nanoscale. First, the spheroid magnetism is a direct fingerprint of nanoscale transformations. Figure 3B shows typical spheroid magnetization curves at different time periods, up to one month, evidencing a massive degradation of the nanoparticles inside the spheroid over time [111]. This is confirmed by electron microscopy observation (Figure 3C), as well as the appearance of multiple ferritin nanoparticles while almost no nanoparticles remain intact for long time periods [111,112]. Unfortunately, this degradation translates into a loss of heat generation at the spheroid level upon magnetic hyperthermia application [112], raising an issue for sequential magnetic hyperthermia application in therapy. However, strategies have emerged to prevent the biodegradation of the magnetic core and, in turn, preserve magnetic hyperthermia functionality. One of them is to shield the magnetic core with an inert gold shell [112,113], and another one is to protect the core with a polymer coating [114]. Alternatively, another strategy could be to seize the capacity that some human cells must biosynthesize magnetic nanoparticles anew from the iron delivered by the degradation of synthetic nanoparticles, thus providing the cells with biogenic non-degradable nanoparticles and long term magnetism [115,116].

## 5. Regulatory Routes to Clinical Approval and Commercial Status

Key to the clinical development of magnetic nanoparticle hyperthermia is the availability of clinically approved, commercially available MNPs. While a profusion of research over the past 20 years has focused on optimizing novel nanoparticles for hyperthermia [117], to date, only a handful of formulations have been approved, and only one for hyperthermia (Table 2).

It should be noted that there is no intrinsic regulatory barrier to the approval of magnetic nanoparticles for clinical use. Indeed, the first iron-oxide formulations were approved in the mid-1990s in Europe, the US and Japan as MRI contrast agents [118]. These particles showed themselves to be generally safe and effective, although most have since been withdrawn as the imaging market moved away from iron oxide agents to favor Gadolinium-based ones.

**Figure 4:**
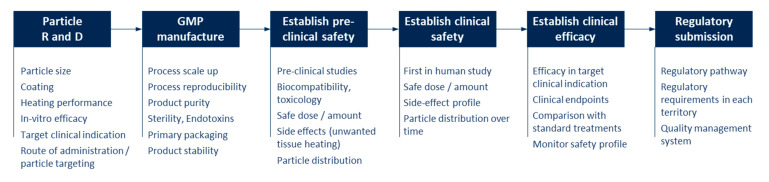
Steps to regulatory approval of MNPs for magnetic hyperthermia.

However, even for MNPs that have been successfully manufactured in the lab and shown promising performance in in vitro and small animal studies, the route from research to regulatory approval and commercialization is complex, expensive and time-consuming, with many aspects to consider at each stage (Figure 4). Undoubtedly this has proven a barrier to the clinical translation of magnetic hyperthermia. Each step requires significant investment, and failure at any stage can lead to delay or discontinuation of the product.

These steps will not be explored in detail here, but common pitfalls include: using preclinical models that do not translate accurately to the clinic; using lab-scale processes that are not readily scalable to volume manufacture; not considering biocompatibility early in the development; product in its final packaging not being physically or chemically stable over desired shelf life, and failing to account for the specific regulatory requirements for the territory where approval is being sought. One area of particular challenge is establishing a particle manufacturing process to GMP standards. Involving a contract manufacturing organization (CMO) with the specialist skillset required can help to reduce the manufacturing risk.

### 5.1. Preclinical Stage

A number of companies have progressed towards addressing manufacturing and quality standards both for the research market and, for some, as a step towards clinical approval (Table 3). The primary focus has been on size distribution, magnetic performance, or coating properties, and only a few make claims to GMP manufacturing, a prerequisite for clinical translation.

Currently available magnetic particles are predominantly iron oxide-based, which is understandable given its history and safety profile. Optimization for hyperthermia may necessitate looking beyond mixed-valence iron oxides [121], but any novel material will need to pass through toxicity screening and full development to deliver a significant advance in clinical use.

### 5.2. Clinical Stage

Each health authority has different sets of requirements for regulatory approval, but the basic elements are similar: In order to achieve approval to sell a magnetic hyperthermia particle product, the manufacturer needs to demonstrate that the product is safe to use, works effectively as a treatment in its chosen clinical indication, and is manufactured under an appropriate quality system to ensure consistent product quality.

**Figure 5:**
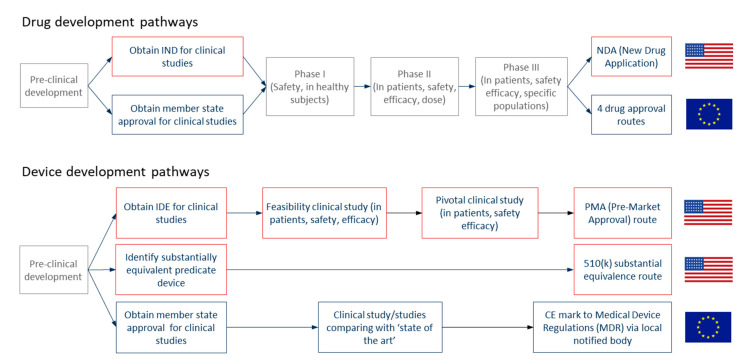
Development and regulatory pathways for drugs and devices in the US and Europe. IDE stands for Investigational Device Exemption, while IND stands for Investigational New Drug Application.

The first question to determine is whether the product is a drug or a device. Each regulatory agency has its own definitions (Table 4), but in general, drugs achieve their intended purpose through chemistry (primarily pharmacology, metabolism) while devices work through physics. A particular challenge of the nanoscale is that the dividing line between “physics” and “chemistry” can be less distinct. Drugs and devices have different regulatory pathways with distinct clinical requirements [122] (Figure 5); hence this determination critically influences the course of the development.

Devices are assigned a class (I, II, or III) depending on the level of product risk, which, in turn, determines the scope of development. The pathway for a lower risk device is likely shorter than a typical drug pathway. Hyperthermia nanoparticle device products are classified as class III in Europe under the medical device regulations (MDR) as they contain nanoscale materials [123]. The US FDA also has classified its first approved nanoparticle device product in class III [124].

#### 5.2.1. The Importance of Primary Mode of Action (PMOA)

The drug/device definitions make clear that the “primary mode of action” (PMOA) or the way in which the “primary intended purpose” is achieved is critical to deciding whether a product is a drug or a device. If the primary way in which the product achieves its intended purpose is through chemical (primarily pharmacological) action or via metabolism, then it is a drug; if it does not, it is a device. In theory, this means that the same compound or substance could be a drug in one indication and a device in another, and this can be seen in the case of IONPs. Feraheme^®^ and Resovist^®^ are approved as drugs because they achieve their PMOA via metabolism or pharmacology; while Nanotherm^®^ and Magtrace^®^ are classified as devices because they achieve their PMOA via non-pharmacological, nonmetabolic means, in this case, magnetic heating and magnetic sensing, respectively. For magnetic hyperthermia, a device PMOA is appropriate if the main intended purpose is achieved through magnetic heating of the nanoparticles, a physical mode of action not involving pharmacology or metabolism. This has proven to be the case for the Nanotherm^®^ particle, which has been approved as a device in Europe [125] and is progressing on a device pathway in the United States.

A secondary mode of action may be included without affecting the determination. For example, a hyperthermia particle may be intended to be metabolized eventually, but only after it has achieved its PMOA of heating. Therefore, the *primary* mode of action would remain as a device.

#### 5.2.2. Drug-Device Combination Product

Where a product uses both drug and device functions to achieve its primary intended purpose, it is classified as a drug-device combination product [128]. A hyperthermia particle that has a targeting function using a receptor (i.e., chemical/pharmacological action), as well as heating, would be classified as a combination product. For combination products in the US, the primary mode of action determines whether the primary regulatory pathway will be drug or device. In Europe, combination products are either regulated as drugs or devices depending on the primary versus ancillary function. For particles combining more than one type of action, the appropriate regulatory pathway will depend on which mode of action is *primary*, and typical elements of both the drug and device pathway are followed, depending on the territory.

### 5.3. Streamlined Development

Any would-be hyperthermia therapy will need to demonstrate clinical safety and efficacy, and for this, there is no short-cut, but there are ways to streamline the overall process. One approach is to use an existing particle in a new way. Such “re-purposing” is the basis for physician-led “off-label” use of therapies. For example, there are a number of clinical studies investigating the use of particles approved for iron-replacement therapy as MRI contrast agents [129]. The rationale is that if a product is shown to be safe in one clinical indication, it will be easier to establish safety in a new one.

The process can also be streamlined by taking into account the requirements of later stages, even in the earliest stages of particle development. Ensuring that manufacturing questions such as toxicity, biocompatibility, stability and process scalability are considered early on can direct the development away from “dead-ends” and save significant cost and time. For example, another nanotechnology, quantum dots, show promise for cancer therapy. However, at first, the most commonly used cores contained cytotoxic cadmium [130]. While in theory, these cores could be coated to minimize cytotoxicity, in practice, establishing long-term safety has proved challenging. Initial consideration of biocompatibility could have accelerated progress towards clinical use by directing effort towards the cadmium-free non-toxic alternatives now being explored [131].

In summary, the challenging later stages of magnetic particle development can be more easily negotiated if, at the early-stages, downstream requirements such as GMP manufacture, clinical safety and the regulatory pathway can be incorporated and used to guide the development. This kind of integrated approach can help hasten a bright future for clinical magnetic hyperthermia.

## 6. Nanotoxicity of Nanoparticles for Magnetic Hyperthermia

The increasing use of magnetic hyperthermia in (pre)clinical settings warrants a proper understanding and careful evaluation of how engineered nanomaterials would be most optimally suited. This concerns both the efficacy of thermal conversion, with maximal heat generation for a minimal number of nanoparticles, as well as a complete lack of any potential toxicity on healthy cells from the patient. To date, various studies have looked into the toxicity of IONPs, mainly driven by their clinical acceptance as contrast agents for MRI [132,133]. The majority of these studies concern in vitro experiments, where cell types of interest are used to evaluate potential toxicity upon exposure to the engineered nanoparticles [134,135]. While more limited, some studies have also been performed in preclinical animal models, mainly mice and rats, to evaluate potential systemic toxicity from exposure to the nanoparticles [136].

However, final conclusions regarding the safety or toxicity of these nanoparticles remain difficult to answer, mainly driven by the wide variety of model systems used, experimental settings and nanoparticle properties [137]. Overall, it is believed that IONPs are fairly safe, up to concentrations of 5 mM of iron [138]. Yet, these early opinions have been revisited by various studies, showing that nanoparticle-specific properties such as size, shape and surface chemistry can have a significant influence on the toxicity of the particles [139]. Overall, toxicity has mainly been linked with cellular uptake levels, where IONPs tend to cause high levels of reactive oxygen species, which, depending on the nature of the cells, can lead to oxidative stress [140]. This, in turn, can manifest itself in different ways, potentially resulting in genotoxicity, diminished stem cell differentiation, neurotoxicity or inflammation [141,142,143,144]. In view of cancer therapy, the sequestration of dextran-coated IONPs by tumor-associated macrophages has been shown to alter macrophage status and promote proinflammatory M1 phenotype [145]. While this is potentially interesting for tumor immunotherapy, the effect of the particles on other macrophages and the induction of inflammatory responses may cause severe side-effects.

As mentioned above, one main problem lies in the seemingly contradicting data available in the literature, which can be narrowed down to experimental variations. Minor modifications to nanoparticle properties can have major implications in view of their biodistribution and toxicity, and, therefore, no general conclusion can ever be made. IONPs also interfere with various classical biochemistry tests, such as the MTT assay [135], and care must be taken to properly design studies with suitable controls or in interpreting data from other studies. For IONPs, one aspect of interest is their biometabolism, where cellular uptake of the nanoparticles results in their lysosomal sequestration. There, the low lysosomal pH and presence of small molecules (e.g., citrate) result in the dissolution of the nanoparticles and the release of ferric ions that are then shuttled into the cytoplasm and become part of the cellular labile iron pool [138]. While this degradation results in proper bioprocessing of the nanoparticles, it will affect the magnetic properties of the nanoparticles in the longer term [146,147]. In the short-term, the kinetics of this degradation must be carefully controlled, as rapid dissolution is linked to excessive ferric ion concentrations present locally that can surpass toxic thresholds [148]. Carefully controlling the degradation kinetics by tuning nanoparticle properties, such as the surface coating, can play a key role in determining the tolerance of the body to such nanoparticles.

In order to further exploit the clinical use of these nanoparticles in hyperthermia applications, it is imperative that the scientific question is properly posed. The question: “are nanoparticles safe” is too generic and can simply not be answered. By rephrasing the sentence into “does formulation x cause any harm when it is used for hyperthermia when administered by y at dose z?”, it defines better the research that needs to be performed in order to promote this field: (1) the exact nanoparticle formulation must be well characterized and described. (2) The route of administration to the patient must be clearly defined. (3) The dose and need for repeated administrations or not must be specified. (4) The ability to perform hyperthermia at its optimal output must be evaluated and compared to current gold standards and state-of-the-art methods. While large sets of literature data are available, the data needed to answer the question above remain scarce. Typical examples include classical toxicity studies, in which nanoparticles are administered systemically by intravenous administration, while for most hyperthermia applications, the nanoparticles are administered locally. IONPs have been studied, but other nanomaterials that are evaluated as hyperthermia mediators with possibly higher therapeutic efficacy are often less commonly studied [149,150]. The biodistribution of the nanoparticles released from the tumor after hyperthermia application would be more interesting to study than systemically administered nanoparticles. The toxicity of the nanoparticles on their own is important, but their effect must also be evaluated after hyperthermia application. It is likely that the surface coating of the nanoparticles has changed due to the generated heat, and this may affect nanoparticle behavior (biodistribution and toxicity) quite drastically. Apart from safety, the therapeutic efficacy must also be demonstrated. While this commonly happens using classical treatment (e.g., doxorubicin), this is often far from reality, and clinically relevant treatments, as well as other novel state-of-the-art therapies, must be used as a comparison (e.g., small molecules, immunotherapy, etc.).

The clinical acceptance of a particular nanoparticle formulation also requires regulatory approval. While guidelines for chemicals to pass clinical trials are rather clear, for nanoparticles, this remains rather vague. Progress has been made, however, and with further optimization and teamwork between experts in the field, the throughput of clinical translation of nanomedicines will only increase. Thus, further advances in the clinical translation of nanotechnologies can be expected in the near future [151]. To date, nanoparticles can either be labeled as a “medical device” or as a “drug”, which has major implications on how regulatory approval for clinical use can be obtained. The label of “medical device” was previously preferred as it involved less lengthy clinical studies and was based on the notion that the nanoparticles themselves did not change or were an active substance but merely a tool by which therapy could be performed. As described in the excellent manuscript by Jones et al. [152], the slow progress in clinical translation is not in itself caused by a lack of regulations but rather a broad gap between theoretical knowledge and practice. This is in part due to the lack of academics involved in setting up the documents regarding safety and efficacy testing of medical devices, resulting in a lack of standardized methods for gathering and presenting data. Efforts to bridge this gap are ongoing, with the setup of the Nanotechnology Characterization Laboratory (NCL) to develop standardized protocols for toxicology, pharmacology and efficacy evaluation of nanomaterials. For most agents, the use of specialized clinical research organizations (CROs) is warranted, who will design and/or perform the preclinical studies required to file an application for clinical studies. While various CROs exist with broad expertise in a wide range of technologies, CROs with expert knowledge on nanoparticle use remains limited. Progress in this domain is expected as the field of nanomedicine keeps expanding, where nanotoxicity and nanomedicine experts can then liaise with, or form part of, regulatory offices and CROs dedicated to enhancing clinical progress in this exciting field.

## 7. Temperature Measuring and Monitoring

### 7.1. Background

Apart from their general application for distance controlled non-contact heating, MNPs provide an excellent opportunity for nano-actuation. For instance, they can be used for selective heating of nano-objects to induce point reactions at the nanoscale. This operation implies the generation of temperature gradients in the nano-object with respect to its surroundings, which can only be determined by thermometers with a spatial resolution also at the nanometre scale. Moreover, the simultaneous use of nanoheaters and nanothermometers could be employed to investigate phenomena of heat transfer at the nanoscale, which is actually an unexplored territory and the object of intense debate, especially concerning intracellular heat transfer [153,154]. The recent development of rare-earth-doped luminescent MNPs has introduced a new field in thermal biosensing, implying less invasive experiments, not only in living cells but also in more challenging small animal models [155,156,157,158]. Joining thermometry and heating at the nanoscale can also be particularly interesting for the development of high-performance non-invasive hyperthermia therapy based on the local heating of specific intracellular sites to provoke cell apoptosis, without the need of overall massive heating of the whole cancer tumor.

The hypothesis of local intracellular hyperthermia arises from the drawbacks of the current strategy of overall tumor heating and a massive injection of MNPs directly into the tumor [159]. Even before being tested, this hypothesis has already been the subject of intense debate [160]. Objections come mostly from standard thermodynamic considerations, which estimate an intracellular concentration of MNPs required to eradicate tumors that is virtually unfeasible [160]. At the center of this debate is the question of whether it is possible to create a sufficiently high-temperature gradient in the vicinity of MNP dispersed in a liquid or intracellular media [154]. Indeed, theoretical thermodynamic calculations predict the formation of negligible temperature gradients in MNPs when subjected to alternating magnetic fields with respect to the bulk [161,162], and a few experimental reports are in agreement with these predictions [163]. However, numerous experimental studies have found substantial local temperature gradients at these conditions that suggest the existence of nanoscale heat transfer phenomena different from macroscopic system behavior [160,164,165,166,167,168,169]. It is thus peremptory for the advancement in the field to dispose of reliable methods for the determination of local temperature in the vicinity of MNPs, and the derivation of thermodynamical models for intracellular heat transfer at the nanoscale.

### 7.2. Luminescence Nanothermometry

Luminescence thermometers are probably the best option for non-contact high spatial resolution thermometry in general [155] and intracellular thermometry in particular [156]. There are several types of luminescent thermometric probes according to their nature: luminescent molecules (organic molecules such as dyes, proteins like green fluorescence protein, GFP), inorganic compounds (quantum dots, Si-dots, nanodiamond, lanthanides) or hybrid molecular or particulate materials. Several temperatures sensing optical properties are also used, such as the intensity of emission, lifetime or polarization anisotropy [157]. Lifetime measurements offer high sensitivity and minimize interferences from other luminophores present in the medium. This is especially true when using lanthanides luminescence probes as they show long lifetimes, eliminating any interference effects from other luminophores. However, lifetime measurements require a sophisticated detection system, and therefore intensity measurements, which can be performed on widely available fluorescence microscopes, are usually preferred. The simplest fluorescence thermometric system is based on single emission intensity measurements, but this system does not yield absolute temperatures, and it is likely to be affected by the concentration of emitters, the intensity of excitation light source and the environment. A way to overcome these problems is to use the ratio of two emissions as the thermometric parameter (ratiometric thermometry), which is far more reliable, especially when the double emission comes from a single source.

There are several ways to implement luminescence nanothermometry into a magnetic nanoheater: the dual-particle approach and the single-particle approach [157]. There are also several kinds of temperature measuring [155,156,157,169]: indirect single temperature value, direct instant, continuous measurements of temperature changes, and in situ ratiometric absolute temperature measurements. In the next section, we will consider the best available options so far for magnetic hyperthermia applications.

### 7.3. Determination of Local Temperature in MNPs

When conveniently endorsed with targeting agents, magnetic NPs have the capacity to selectively penetrate the membrane of cancer cells. Providing that they can be directed to specific intracellular organelles, the heat generated by these NPs can be used to induce local damage in these organelles originating cell apoptosis without the need of increasing the temperature of the whole cell and thus substantially reducing the amount of MNPs necessary to kill the cells. To verify this hypothesis, it is necessary to check whether the heat generated by the MNPs is sufficient to maintain a temperature gradient between the targeted organelle against the heat conduction to the cytoplasm and the extracellular matrix. Moreover, this concept of local hyperthermia therapy with AC fields requires the use of MNPs with a high SLP at conditions compatible with in vivo treatment. Dual MNP-thermometer nanoparticle systems lack precision as the temperature is measured at a distance from the heater [167]. Single-particle systems based on the detachment of fluorophores from the MNP shell above certain temperature values have been used to obtain temperature gradients of about 45 °C [166] and 8 °C [167] in the vicinity of MNPs under AC magnetic field induction. However, these methods require post-analysis of the medium and therefore cannot be used for temperature monitoring. The ideal solution is to incorporate in a single MNP a thermometric probe that yields instant information of the local absolute temperature of the MNP during the application of the alternating magnetic field. There are two possible ways: decorating the MNP surface with molecular thermometers or coating it with a solid thermometric shell. The second case would offer better chemical stability against the complex intracellular environment, but it is still at the stage of finding an adequate inorganic thermometric coating for MNPs. Molecular luminescence probes may suffer from luminescence quenching and bleaching effects produced by chemicals in the environment. Therefore, the real challenge in the design of this type of probes is to shield them against the action of the complex biological environment to preserve their optical thermometric properties.

**Figure 6:**
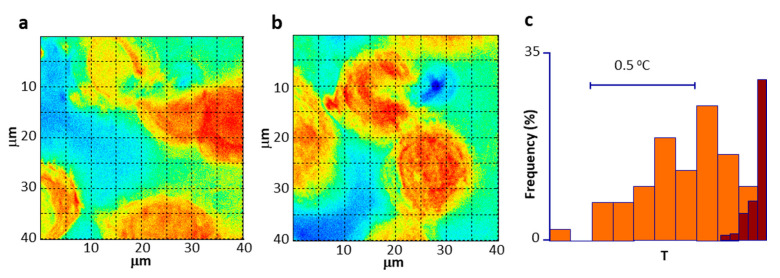
Thermal images of MNPs internalized in cells, (**a**), before, and (**b**), during irradiation with an AC magnetic field; (**c**), average temperature shift upon the application of an AC magnetic field.

As introduced in Section 7.2, several types of materials have been proposed as temperature nanoprobes [156], some of them have been even used for intracellular thermometry [170], i.e., ER thermo yellow [171], but only a few have been actually used in local magnetic hyperthermia studies [170]. For instance, reports of a 15 °C temperature gradient were reported on Mn-ferrite NPs attached to the membrane of HEK 293 cells 15 s after the application of AC fields using DyLight549 as a thermometric probe [164]. Advances in this case also include the in situ measurement of local temperature gradients using rhodamine [172] and lanthanide luminescence complexes [169]. The emission lifetime of GFP has also been used to report large local temperatures of 85 °C, although they were estimated by extrapolation of calibration curves obtained at much lower temperatures. The probes were not attached to the heathers, but independently spread inside the cells. Using a double particle approach, consisting of MNPs and upconversion nanoparticles embedded in a silica matrix, and ratiometric temperature determination, gradients of about 20 °C were reported on nanoparticles suspended in a liquid after a 5 min exposure to AC fields [168]. Ongoing intracellular experiments in our lab using similar nanoprobes have yielded quite promising results on 2D-temperature imaging of cells containing MNPs during hyperthermia treatment (Figure 6). Most of the thermometric probes described so far are based on visible light, lacking the necessary tissue penetration for body temperature monitoring, and therefore can only be useful in cell culture experiments. However, the field is also moving towards the development of infrared deep tissue thermometry systems [173]. This option is possible in the case of optical hyperthermia, where single nanoparticles with a heating and temperature measurement properties are already available [174]. Infrared luminescence temperature probes can also be possibly coupled to magnetic heating nanoparticles.

We can reasonably expect that in a few years, we would dispose of several types of MNPs incorporating local temperature probes, both molecular and solid nature, that can yield reliable data on the local temperature gradient generated by MNPs exposed to alternating magnetic fields that can be used for the development of less invasive, more efficient and more selective advanced magnetic hyperthermia treatments.

## 8. Treatment Planning and Dosimetry

Treatment planning—which reproduces the dose needed to destroy the tumors preserving as much healthy tissue as possible—for many hyperthermia modalities have benefitted from the developments made in radiotherapy [175,176,177,178,179]. In the particular case of magnetic hyperthermia, so far, there is only one commercially available system for the purpose developed by MagForce, the NanoPlan^®^ (formerly known as HyperPlan^®^) [180]. The typical workflow of a hyperthermia treatment planning (HTP) tool starts with a 3D model of the region of interest that is built from computerized tomography (CT), or MRI scans from patients; then, electromagnetic fields, SAR and temperature distributions are computed by solving Maxwell’s and bioheat equations, respectively, under the appropriate boundary conditions. Regarding the virtual models used, a more or less extensive collection is available throughout the literature—see, for instance, review [181]. The use of these patient-based models—despite adding complexity to the HTP process—has been demonstrated to improve the estimation of SAR and temperature patterns compared to homogeneous phantom-based models [182]. Many physical models used for simulating the interaction of the applied electromagnetic fields with tissues have been proposed [183,184,185,186,187,188], mainly by deriving from fundamental equations, like Maxwell’s equations—for calculating electromagnetic fields and SAR—and Penne’s bioheat equation—for temperature distributions. Unfortunately, modeling magnetic hyperthermia within the context of this body of knowledge still proves difficult mainly due to an important challenge: to couple the different size scales—from 3D down to 1D, where the relevant magnetic and heating phenomena stem from—involved in the multiple physical phenomena converging in this therapy, namely fluid dynamics, heat exchange, and electromagnetic interaction. Due to its inherent complexity, this is a longstanding problem that needs to be addressed in the short-term to move towards wider clinical adoption of magnetic hyperthermia. A unified magnetic hyperthermia theory should be made possible in the near future, taking advantage of 3D-1D coupling strategies based on topological model reduction [189].

**Figure 7:**
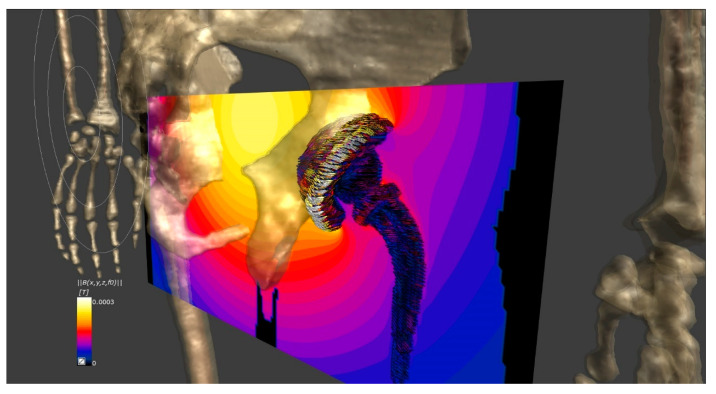
Calculated field and temperature patterns in and around the hip implant of a prospective patient of magnetic hyperthermia to treat a prostate tumor.

New HTP systems based on convolutional neural networks and deep-learning techniques are being tested in a new clinical trial focused on the treatment of locally advanced pancreatic ductal adenocarcinomas [2], allowing to obtain a 3D model for each patient instead of relying on the standard ones already available to perform the simulations. The main benefit of the HTP is that all cases can be studied beforehand, even those that match with any of the exclusion criteria, thus evaluating their risk and finally ruling out or not their suitability for the treatment. One of the exclusion criteria for prospective participants is that no implant-bearing patients are allowed, which may represent a proportion as high as 60% of the initial cohort. The reason for this is that part or totally metallic implants may undergo noticeable heating during the therapy upon being exposed to the magnetic field needed to excite the MNPs (Figure 7), even inducing tissue damage if not properly controlled. Very recent results show how the implant heating process takes place in different indications, and different ways to recover a sizeable percentage of the initially excluded patients have been proposed [190,191].

Future big improvements in treatment planning for magnetic hyperthermia are on their way, coming from the hand of specific emerging nano-enabled diagnostic techniques, like the case of magnetic particle imaging (MPI). The latter specifically adds what treatment planning in magnetic hyperthermia is currently lacking, namely the precise quantification and spatial location of the nanoparticles inside the body depending on the administration means and the physical properties of the tissues involved [192]. This feature allows for a preliminary quality check of nanoparticle installation inside tumors, basing the predictions on the number of nanoparticles that have actually reached the target and not the rough estimations made from the expected spread of the injected volume. In addition, the possibility of a real-time follow-up of the post-injection fate of nanoparticles is crucial to correct the initial conditions for simulating subsequent treatment sessions. Considering that the development of MPI scanners for humans is currently moving on [193,194,195], the first results from incorporating it into the current HTP methodologies may be seen in about two years.

Another significant step-up in HTP for magnetic hyperthermia could be achieved in the near future by combining the existing technology with *radiomics*. The latter comprises computer-assisted medical image analysis with dedicated algorithms for feature extraction—much benefitted from the boom of artificial intelligence—providing spatial and temporal information that is not attained by data from -omics [196,197]. Radiomics mainly nurtures from computer tomography and magnetic resonance imaging data, but applying its principles to MPI may bring an unprecedented degree of accuracy to HTP in magnetic hyperthermia.

## 9. Further Evolution into Theranostics: Combining Magnetic Hyperthermia and MPI

Combining therapy and diagnostic imaging, or theranostics has been an active area of research across numerous biomedical fields over the past decade. For example, theranostics is an established approach in nuclear medicine whereby therapy and diagnostic imaging are performed using the same molecule or similar molecules. Theranostic agents enable simultaneous assessment of clinical status, treatment, and confirmation of treatment dose. In this section, we discuss a new theranostic approach that combines MPI and localized magnetic hyperthermia. Together, these two technologies enable mapping of a MNP distribution, prescribing a heating dose, and then carefully applying heat per the prescribed heat dose to a local area.

**Figure 8:**
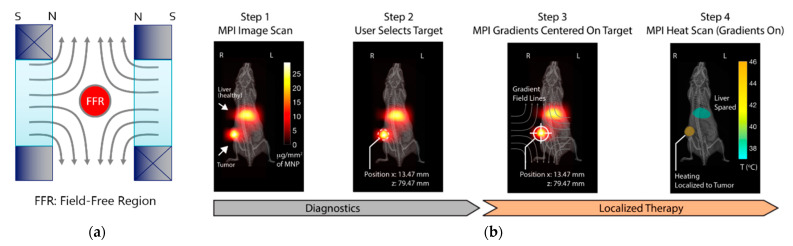
(**a**) MPI uses a selection field to localize the nanoparticle signal. The diverging magnetic field lines produce a unique FFR in the center. By varying the applied magnetic field, the FFR can be translated across the sample; (**b**) workflow of an MPI-directed localized magnetic hyperthermia therapy for solid tumors. Following tracer administration, MPI was performed to identify tumor location and size and to identify tracer uptake in healthy tissue such as the liver. The measured target dose and off-target areas of risk are then used to optimize magnetic hyperthermia treatment planning. Follow-up scans can be performed to assess response to therapy over time. Reprinted (adapted) with permission from [212].

MPI is an emerging imaging technology that directly quantitates MNP concentration in tissue. MPI is tracer-based and produces positive contrast images, analogous to nuclear medicine or optical. The signal is directly detected from the nanoparticle tracers and has the advantage of being linearly quantitative without tissue attenuation. The physics that underlie the signal generation and image formation can be understood using classical physics. An MPI system produces a strong magnetic field gradient containing a field-free region (FFR)—a region where the magnetic field is approximately zero (Figure 8a). MNPs in the FFR are magnetically unsaturated and produce a signal in a receiver coil, while saturated superparamagnetic iron oxide nanoparticles (SPIOs) outside the FFR produce no signal. Images are produced by raster scanning the FFR across the subject. First published in 2005 [198], the field has grown rapidly, and there are now commercially available preclinical MPI systems [199,200], and clinical-scale MPI systems are under development [193,194].

To date, most MPI has been performed to non-invasively image the distribution and quantity of MNPs in murine models. Tumors can be detected by the passive accumulation of tracers through the enhanced permeability and retention effect [201], by the uptake of phagocytic tumor-associated macrophages [202], or through the targeted use of functionalized and targeted tracers [203,204]. Novel nanoparticles enable new capabilities for MPI systems. Song et al. demonstrated the synthesis of multimodal FeCo nanoparticles for imaging with near-infrared, MPI, MRI, and photoacoustic techniques and therapeutic properties with photothermal and magnetothermal systems [205].

As previously discussed, magnetic hyperthermia for activation of MNPs offers considerable potential for numerous biomedical applications, especially in the clinical treatment of cancers. Magnetic hyperthermia relies on the delivery of MNPs to tumors followed by the application of AC fields, causing local heating of tissue. The killing of tumor cells occurs either directly or by enhancing the cytotoxic effects of radio, immune, or chemotherapy [206]. Magnetic hyperthermia can be performed anywhere in the body since AC fields penetrate tissue without attenuation. Human clinical trials have demonstrated the benefits of magnetic hyperthermia for prostate cancer [207]; and, overall survival benefits with radiotherapy in recurrent glioblastoma resulted in European regulatory approval in 2010 [208]. Despite its demonstrated effectiveness, current magnetic hyperthermia implementations are limited by the accumulation of MNPs away from the lesion of interest, the inability to visualize MNP distribution during treatment, and limited ability to monitor tissue temperature [209]. These limitations result in poor MNP heating control, reduced therapeutic effect, and increased collateral damage.

Recent advances have demonstrated that applying the strong gradient magnetic field used for imaging in MPI during magnetic hyperthermia enables localized magnetic hyperthermia, which can help overcome many of the limitations faced by traditional magnetic hyperthermia technologies. Localized magnetic hyperthermia allows researchers to exert spatial control over which MNPs are heated with millimeter-scale resolution [210,211,212]. Tay et al. demonstrated the use of a magnetically localized magnetic hyperthermia system to spatially localize heat deposition to a tumor while avoiding heat deposition to the healthy liver [212]. Further, the heat dose can be optimized in real time by moving, expanding, or contracting the size of the FFR, changing the heating region accordingly.

Further improvements in the accuracy of treatment can be made by combining both MPI and localized magnetic hyperthermia for a complete theranostic workflow that combines imaging, treatment prescription, and application of therapy (Figure 8b). The important linking factor across the two technologies is the availability of MNPs that are both visible in MPI and capable of being heated via magnetic hyperthermia [209,213]. In addition to nanoparticle biodistribution, the MPI signal contains information on the MNP’s microenvironment and a combined MPI, and magnetic hyperthermia can enable non-invasively measuring temperature during heating [212,214,215]. Accurate temperature measurement is particularly important to prevent over- and under-treatment of tumors whether the tissue is being heated to apoptosis or as an immune-stimulating adjuvant therapy.

The combination of MPI, magnetic hyperthermia and localized magnetic hyperthermia opens new directions for both research and clinical treatment. In research, new applications are possible such as a novel, magnetic hyperthermia actuated nanotherapeutics capable of the localized release of drugs or expression of heat-sensitive genetic promotors. Ultimately, as MPI and localized magnetic hyperthermia are clinically translated, we can expect workflow similar to X-ray/CT guided Radiation Therapy.

## 10. Standardization of Magnetic Colloids for Magnetic Hyperthermia

### 10.1. General Aspects

The need for standardization of MNPs for magnetic hyperthermia forms part of a much broader demand for reliable, reproducible, stable and well-characterized nanostructured magnetic materials for use in emerging applications in medicine and other highly demanding sectors. However, standardization also includes the provision of well-defined and reproducible measurement methods for the characterization of the materials. These methods are required to reliably determine magnetic (and structural) parameters of reference materials and of the MNPs employed in magnetic hyperthermia. This standardization need, the related science of MNPs and their characterization have been previously explored in some detail [216,217]. In the following subsections, the key tools available to aid in realizing the standardization of MNPs for hyperthermia therapy will be described. Combined, the steps described here form a roadmap for the future realization of a measurement and standardization infrastructure for MNPs for hyperthermia therapy.

### 10.2. Validating Metrological Traceability at Key Laboratories

While not a realistic goal to be achieved at every measurement laboratory in the characterization community, it is of great importance that the metrological traceability to SI units of at least some measurement laboratories is demonstrated for each involved characterization technique. National metrology institutes are typical locations for this work to be undertaken, as they combine the expertise, equipment and resources necessary to undertake this critically important work. This is the only possible way to truly verify that measurements are accurate, quantitative and coherent, with verified uncertainties. European metrology institutes already have a generalized framework in place for undertaking this kind of work, which has previously been applied to other areas of industry with great success. Hence, far, none of the magnetic measurements for MNPs have been metrologically validated on calibrated instruments, while some preliminary work on other non-magnetic characterization techniques has already been undertaken [218]. Establishing metrological traceability to SI units for key characterization techniques is necessary to validate MNPs for hyperthermia therapy. This work is of utmost importance to achieving standardization and deserves the highest priority. The early involvement of the relevant end-user industry figures will ensure the long-term success of the validation work.

### 10.3. Interlaboratory Ring Comparisons to Harmonize Measurements

In addition to establishing metrological traceability at individual key laboratories, developing an understanding of the overall level of measurement agreement, accuracy and repeatability across the entire measurement community must also be achieved. Ring comparisons (also called interlaboratory comparisons) are the proven tool for undertaking this type of work. By distributing identical sample sets to multiple laboratories and monitoring the findings, a huge amount of information can be obtained. By changing variables such as the use of in-house or centrally developed operating procedures, the extent of uncertainties introduced by measurement apparatus, measurement procedures and analysis methods can be isolated, understood and mitigated.

To date, ring comparisons have been performed for various nanoparticle parameters relevant to hyperthermia characterization, including DLS [219], static magnetization, SLP/ILP and AC susceptibility measurements. Alarmingly, these studies have revealed a strong need for further harmonization to achieve interlaboratory agreement. Regular ring comparisons are the only realistic way to monitor progress in the harmonization of nanoparticle characterization techniques and reveal the extent of interlaboratory variations.

### 10.4. Development of Reference Materials

To date, no verified or accepted reference materials exist for any of the properties of MNPs relevant for magnetic hyperthermia therapy. Reference material is characterized by being homogeneous and stable with respect to a certain material characteristic: this can be a physical quantity like the initial magnetic susceptibility, but it can also be a performance characteristic like colloidal stability. For reference materials with defined physical properties, it is desirable to have a certified measurement of this property (made with calibrated instruments in a manner that is metrologically traceable to SI units), together with an accurately rendered uncertainty. Materials for which this type of validated measurement has been conducted, and which are validated as being stable, are called certified reference materials (CRM). CRMs are indispensable for verifying the accuracy and temporal stability of individual measurement equipment, for achieving successful ring comparisons and accurately assessing in-house quality assurance systems.

At present, large manufacturers of MNPs use their own in-house reference materials to verify their magnetic properties measurements. Smaller companies and academic institutes typically rely on other commercial MNP products as quasi-reference materials while lacking detailed knowledge about the actual batch-to-batch or temporal stability of the material. The development of validated CRMs for magnetic nanoparticle properties will benefit all levels of industry and research and is vital to the large-scale manufacturing of medical-grade MNP materials suitable for magnetic hyperthermia therapy. The development of CRMs for this purpose, therefore, deserves to be the focus of significant effort in the coming years.

### 10.5. Calibration and Certification of Measurement Devices and Services

Industrially accepted testing laboratories typically operate under strict systems for quality control and management. Guidance to inform best practice in developing these systems for biomedical products is available from ISO [220]. Laboratories that adhere to these quality standards can gain accreditation from national bodies that operate under mutual recognition agreements provided by the International Laboratory Accreditation Cooperation (ILAC) to ensure that laboratories produce results that are of a known quality that is regularly monitored. The level of standardization in the MNP manufacturing and characterization communities is not yet sufficiently advanced for this step to be possible. The requirements laid out in points 10.2 to 10.4 must be realized first. Given the economic importance of MNPs and technologies which rely on them, it is in itself a striking finding that not one laboratory in the world can currently issue accredited test certificates for the hyperthermia performance of MNPs or related characteristics.

### 10.6. Development of European and International Standards Documents

Laying arguably at the pinnacle of the standardization mountain, document standards issued by ISO, CEN (the European Committee for Standardization) or other national or international bodies present in clear terms the agreed best practices for a diverse variety of industries. Until very recently, no document standards relating to the definition or measurement of the magnetic properties of nanoparticles existed. In the last few years, work has begun on a new series of ISO standards to address this need. The ISO 19,807 series presently consists of one published guidance document [32] detailing terminology and appropriate measurement techniques, with a second application-specific document specifying the needs for nanoparticles destined for nucleic acid extraction currently being prepared [221]. In ISO 19807-1, magnetic hyperthermia is defined as the process where a time-varying magnetic field of frequency *f* and amplitude *H*_0_ results in a temperature T increase of a magnetic nanosuspension. Furthermore, the document contains a definition of *SLP* as being the heating power of a magnetic nanosuspension per unit mass in response to a time-varying magnetic field of frequency *f* and amplitude *H*_0_. In this definition, the unit mass can relate to the whole nanosuspension, to the solid content or to other compartments of the nanosuspension. It should be indicated which mass is used in reporting the result of a specific loss power measurement. In addition to the *SLP*, also the *ILP* may be reported, which is given by Equation (1):

(1) $$ILP=SLP\;/\;\left(f\cdot H_{0}^{2}\right)$$

When reporting the *SLP* or *ILP* parameter, also the initial temperature of the magnetic nanosuspension before the heating, as well as frequency *f* and amplitude *H*_0_ of the excitation field, should be indicated. These are definitions of the governing hyperthermia parameters of an MNP suspension. Hence, far, there is no specification for standardized measurements, which remains a task of the future. In addition, issues like MNP concentration and properties of the suspending fluid are not considered in these definitions.

As future progress is made in addressing the standardization tasks detailed in point 10.2 to 10.4, opportunities to develop new document standards to detail the best practices for reference material preparation, conducting specific characterization measurements and other application-specific documents are expected to develop. By engaging with their national standardization institutes, stakeholders from both industry and academia can take part in this ongoing effort and influence the final agreements in best practice for magnetic nanoparticle products.

For the standardization of MNPs for magnetic hyperthermia, validated metrology of the heating properties of MNP is a necessary prerequisite. Key laboratories at National Metrology Institutes and other expert groups should demonstrate their proficiency in *SLP* measurements via interlaboratory comparisons using reference materials. Harmonized measurement procedures for *SLP* measurements on calibrated devices under strict quality assurance should be codified in international document standards. The work to implement the elements of MNP hyperthermia metrology is only at the beginning. Since the first clinical studies on magnetic hyperthermia-based cancer therapies are ongoing, it is of great importance to soon achieve a reliable and reproducible quantitative assessment of the physical properties of the involved MNPs. This will not only improve the safety of the patients but also lead to better opportunities for MNP manufacturers and hyperthermia equipment suppliers.

## Figures and Tables

**Table 1:** MNP parameters and the corresponding characterization techniques. Adapted from [15,16]. See list of acronyms at the end of the document.

**Structural Properties**	
Particle, core and aggregate size	TEM, XRD, DLS, NTA, SAXS, HRTEM, SEM, AFM, EXAFS, FMR, DCS, MALDI, NMR, TRPS, EPLS, magnetic susceptibility
Morphology	TEM, HRTEM, AFM, EPLS, FMR, 3D-tomography
Elemental-chemical composition	XRD, XPS, ICP-MS, ICP-OES, SEM-EDX, NMR, MFM, LEIS
Crystallinity	XRD, EXAFS, HRTEM, electron diffraction, STEM
Structural defects	HRTEM, EBSD
Chemical state–oxidation state	XAS, EELS, XPS, Mössbauer
Ligand-binding, surface composition	XPS, FTIR, NMR, SIMS, FMR, TGA, SANS
**Colloidal Properties**	
Hydrodynamic and aggregate size	NTA, DLS, DCS, UV-vis, SEM, TEM, Cryo-TEM
3D visualization	3D-tomography, AFM, SEM
MNP charge	Zeta potential, EPM
Element concentration	ICP-MS, UV-vis, RMM-MEMS, PTA, DCS, TRPS
**Magnetic Properties**	
Quasi-static magnetization properties	SQUID, VSM, Mössbauer, MFM, FMR, XMCD,
Dynamical magnetization properties	AC susceptometry and magnetometry, magnetorelaxometry, magnetic particle spectroscopy
Magnetic losses	AC calorimetry, AC susceptometry and magnetometry

**Table 2:** Commercially available clinically approved IONPs.

Name (Generic)	Manufacturer	Approved Indication	Approval Date	Regulatory Classification
Feraheme^®^ (ferumoxytol)	AMAG Pharmaceuticals	Iron deficient anemia in chronic kidney disease	2009 (FDA) ^a^	Drug
Resovist^®^ (ferucarbotran)	FUJIFILM RI Pharma	MRI imaging agent for liver	2002 (PMDA) ^b^	Drug
Nanotherm^®^	MagForce	Hyperthermic treatment of glioblastoma	2010 (CE mark)	Device (class III)
Magtrace^® c^	Endomag	Sentinel lymph node biopsy for cancer staging	2011 (CE mark) 2018 (FDA)	Device (class III) Combination Product (class III)

^a^ Approved by the EMA as Rienso^®^ in 2012, but withdrawn in 2015 [119]; ^b^ approved by the EMA in 2001, but European distribution ceased in 2009 [120]; ^c^ formerly known as Sienna+^®^.

**Table 3:** Selection of commercially available research-grade MNPs.

Manufacturer	Name(s)	Development Stage
chemicell GmbH	fluidMAG and nano-screenMAG	Research
Creative Diagnostics	Various product codes	Research
Imagion Biosystems	PrecisionMRX^®^	Research
Liquids Research	HyperMAG™	Research
Magnetic Insight	VivoTrax™, VivoTrax Plus™	Preclinical sterile
micromod Partikeltechnologie GmbH	nanomag^®^, Perimag^®^, Synomag^®^ and others	GMP manufacturing available
Nanopartz	Various product codes	GMP manufacturing available
nanoPET Pharma	FeraSpin™	Preclinical sterile
nanoTherics	HyperMAG^®^	Research
NNCrystal	Various product codes	Research
NVIGEN	MaxVigen™	Research
Ocean NanoTech	Various product codes	Research
Resonant Circuits	RCL-01	Preclinical sterile
SPL Medical	Ferrotran	Undergoing human clinical trials

**Table 4:** EU and US regulatory authority definitions of drug and medical device (emphasis added).

Regulatory Authority	Drug	Medical Device
European Union [126]	Any substance or combination of substances having properties for treating or preventing disease in human beings or; may be used in or administered to human beings with view to restore, correct, modify physiological function ***by exerting a pharmacological, immunological or metabolic action, or to making a medical diagnosis.***	Any instrument, apparatus, appliance, material, software, or other article […] to be used in humans for the purpose of: diagnosis, prevention, monitoring, treatment or alleviation of disease, diagnosis, monitoring, treatment, alleviation of or compensation for an injury/handicap investigation, replacement, modification of the anatomy; control of conception ***and which does not achieve its principal intended action in or on the human body by pharmacological, immunological or metabolic means, but which may be assisted in its function by such means.***
FDA [127]	(A) articles recognized in the official United States Pharmacopoeia […]; and (B) ***articles intended for use in the diagnosis, cure, mitigation, treatment, or prevention of disease in man or other animals*****; and (C) *articles (other than food) intended to affect the structure or any function of the body of man or other animals***; […]	an instrument, apparatus, implement, machine, contrivance, implant, in vitro reagent, or other similar or related article, including any component, part, or accessory, […] ***which does not achieve its primary intended purposes through chemical action within or on the body of man or other animals and which is not dependent upon being metabolized for the achievement of its primary intended purposes.***

## Data Availability

Not applicable.

## Abbreviations

AFM	Atomic force microscopy
AGM	Alternating gradient magnetometery
Cryo-TEM	Cryogenic transmission electron microscopy
DCS	Differential centrifugal sedimentation
DLS	Dynamic light scattering
EBSD	Electron backscatter diffraction
EDX	Energy dispersive X-ray spectroscopy
EELS	Electron energy loss spectroscopy
EXAFS	Extended X-Ray absorption fine structure
EPM	Electrophoretic mobility
EPLS	Elliptically polarized light scattering
FMR	Ferromagnetic resonance
FTIR	Fourier Transform Infrared Spectroscopy
HRTEM	High resolution transmission electron microscopy
ICP-OES	Inductively coupled plasma optical emission spectrometry
ICP-MS	Inductively coupled plasma mass spectrometry
ICP-AES	Inductively coupled plasma atomic emission spectroscopy
ILP	Induced loss power
IONPs	Iron oxide nanoparticles
LEIS	Low-energy ion scattering
MALDI	Matrix-assisted laser desorption/ionisation
MH	Magnetic Hyperthermia
MFM	Magnetic force microscopy
MNPs	Magnetic Nanoparticles
MRI	Magnetic resonance imaging
NMR	Nuclear magnetic resonance
NTA	Nanoparticle tracking analysis
PTA	Particle tracking analysis
RMM-MEMS	Resonant mass measurement microelectro-mechanical system
SANS	Small angle neutron scattering
SAR	Specific absortion rate
SAXS	Small angle X-ray scattering
SEM	Scanning electron microscopy
SEM-EDX	Scanning electron microscopy - Energy dispersive X-ray spectroscopy
SIMS	Secondary ion mass spectrometry
SLP	Specific loss power
SOP	Standard operating procedure
STEM	Scanning transmission electron microscopy
SQUID	Superconducting quantum interference device
TEM	Transmission electron microscopy
TGA	Thermogravimetric analysis
TRPS	Tunable resistive pulse sensing
UV-vis	Ultraviolet–visible spectroscopy
VSM	Vibrating sample magnetometry
XRD	X-Ray diffraction
XMCD	X-Ray magnetic circular dichroism
XPS	X-Ray photoemission spectroscopy
